# Tumor Biomechanics Quantified Using MR Elastography to Predict Response to Neoadjuvant Chemotherapy in Individuals with Breast Cancer

**DOI:** 10.1148/rycan.240138

**Published:** 2025-02-14

**Authors:** Aaditya P. Sinha, Patriek Jurrius, Anne-Sophie van Schelt, Omar Darwish, Belul Shifa, Giacomo Annio, Zhane Peterson, Hannah Jeffery, Karen Welsh, Anna Metafa, John Spence, Ashutosh Kothari, Hisham Hamed, Georgina Bitsakou, Vasileios Karydakis, Mangesh Thorat, Elina Shaari, Ali Sever, Anne Rigg, Tony Ng, Sarah Pinder, Ralph Sinkus, Arnie Purushotham

**Affiliations:** ^1^School of Cancer and Pharmaceutical Sciences, King’s College London, London, United Kingdom; ^2^Breast Unit, Guy’s and St Thomas NHS Foundation Trust, Guy’s Hospital, Great Maze Pond, London SE1 9RT, United Kingdom; ^3^School of Biomedical Engineering and Imaging Sciences, King’s College London, London, United Kingdom; ^4^LVTS, Inserm U1148, University Paris Diderot, Paris, France; ^5^Department of Radiology, Guy’s and St Thomas NHS Foundation Trust, London, United Kingdom; ^6^Breast Unit, King’s College Hospital NHS Foundation Trust, London, United Kingdom

**Keywords:** Breast Cancer, MR Elastography, Neoadjuvant Chemotherapy, Dynamic Contrast-enhanced MRI

## Abstract

**Purpose:**

To evaluate the ability of MR elastography (MRE) to noninvasively quantify tissue biomechanics and determine the added diagnostic value of biomechanics for predicting response throughout neoadjuvant chemotherapy (NAC).

**Materials and Methods:**

In this prospective study (between September 2020 and August 2023; registration no. NCT03238144), participants with breast cancer scheduled to undergo NAC underwent five MRE scans at different time points alongside clinical dynamic contrast-enhanced MRI (DCE MRI). Regions of interest were drawn over the tumor region for the first two scans, while for the post-NAC scan, the initial pre-NAC tumor footprint was used. Biomechanics, specifically tumor stiffness and phase angle within these regions of interest, were quantified as well as the corresponding ratios relative to before NAC (tumor-stiffness ratio and phase-angle ratio, respectively). Postsurgical pathologic analysis was used to determine complete and partial responders. Furthermore, a repeatability analysis was performed for 18 participants.

**Results:**

Datasets of 41 female participants (mean age, 47 years ± 12.5 [SD]) were included in this analysis. The tumor-stiffness ratio following NAC decreased significantly for complete responders and increased for partial responders (0.76 ± 0.16 and 1.14 ± 0.24, respectively; *P* < .001). The phase-angle ratio after the first cycle of the first NAC regimen compared with before NAC predicted pathologic response (1.23 ± 0.31 vs 0.91 ± 0.34; *P* < .001). Combining the tumor stiffness ratio with DCE MRI improved specificity compared with DCE MRI alone (96% vs 44%) while maintaining the high sensitivity of DCE MRI (94%). Repeatability analysis showed excellent agreement for elasticity (repeatability coefficient, 8.3%) and phase angle (repeatability coefficient, 5%).

**Conclusion:**

MRE–derived phase-angle ratio and tumor stiffness ratio were associated with pathologic complete response in participants with breast cancer undergoing NAC, and a combined DCE MRI plus MRE approach significantly enhanced specificity for identification of complete responders after NAC, while maintaining high sensitivity.

**Keywords:** Breast Cancer, MR Elastography, Neoadjuvant Chemotherapy, Dynamic Contrast-enhanced MRI

*Supplemental material is available for this article.*

Clinical trials registration no. NCT03238144

Published under a CC BY 4.0 license.

## Graphical abstract

**Figure:**
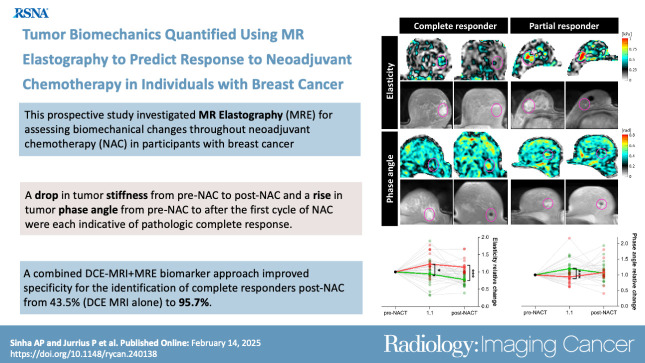

**Summary**

Assessment of tumor biomechanics through MR elastography improved the identification of complete responders among individuals with breast cancer undergoing neoadjuvant chemotherapy.

**Key Points**

■ In individuals who underwent MR elastography in addition to dynamic contrast-enhanced (DCE) MRI throughout neoadjuvant chemotherapy (NAC) for breast cancer, a drop in the tumor-stiffness ratio from prior to NAC to after NAC was indicative of pathologic complete response (complete responders, 0.76 ± 0.16 [SD]; partial responders, 1.14 ± 0.24; *P* < .001).
■ A combined DCE MRI and MR elastography biomarker approach improved specificity for the identification of complete responders following NAC from 44% (DCE MRI alone) to 96%.
■ A rise in biomechanical tumor phase angle ratio from before NAC to the first cycle of NAC was associated with pathologic complete response (complete responders, 1.23 ± 0.31; partial responders, 0.91 ± 0.34; *P* < .001).

## Introduction

Breast cancer is a common disease globally, with 2.3 million diagnoses annually and a lifetime risk of one in seven women in the United Kingdom (1,2). The high prevalence rate makes it the most common cancer in women and the second most common cause of cancer death for women globally (3). Neoadjuvant chemotherapy (NAC) is the primary systemic therapy to downsize solid tumors, after which most patients undergo (breast-conserving) surgery. Currently, the residual cancer burden (RCB) scoring system at final histopathologic analysis is used to predict disease recurrence and survival rates. However, this is not applicable during ongoing treatment with NAC (4). Throughout therapy, breast MRI with dynamic contrast enhancement (DCE) is used as the current reference standard in assessing response clinically, with a sensitivity of typically 80% and a specificity ranging from 37% to 97% (5–8). Additionally, radiologic vacuum-assisted biopsies have been used to assess pathologic complete response (pCR). However, using a 14-gauge needle may not be a reliable predictor of pCR (9–11), with different studies reporting false-negative rates as high as 42% due to sampling errors (9). Current research has focused on de-escalation in breast cancer treatment to potentially avoid surgery and change therapy early for nonresponders (12). Thus, a method for accurate monitoring of response is of critical importance.

One promising avenue for assessing response is to investigate tumor biomechanics (13,14). It is known that tissue angiogenesis, lymphangiogenesis, hypoxia, and inflammation all promote tumor aggression, which exerts mechanical forces on the tumor and its microenvironment (15–17). Furthermore, tissue mechanics are modulated by a solid tumors’ high interstitial pressure, which impacts hypoxia, metastatic propensity, mortality, and treatment outcome (18). It has been shown that invasive regions exhibit an elevated mean stiffness primarily due to an increase in collagen deposition (19). Consistently, unconfined compression analysis shows that tumor stiffness is associated with aggressive cancers (20,21). Therefore, gauging tumor biomechanics noninvasively could be useful for assessment of therapy efficacy.

MR elastography (MRE) is an imaging modality that uses propagating mechanical shear waves to enable noninvasive quantification of tissue biomechanics (22) and has already demonstrated promise in the characterization of breast lesions (23–25). Hence, this study aims to demonstrate *(a)* the feasibility of using MRE as part of clinical breast cancer NAC and *(b)* to investigate the added diagnostic value of biomechanics for predicting response throughout NAC.

## Material and Methods

### Study Design and Participants

**Figure 1:**
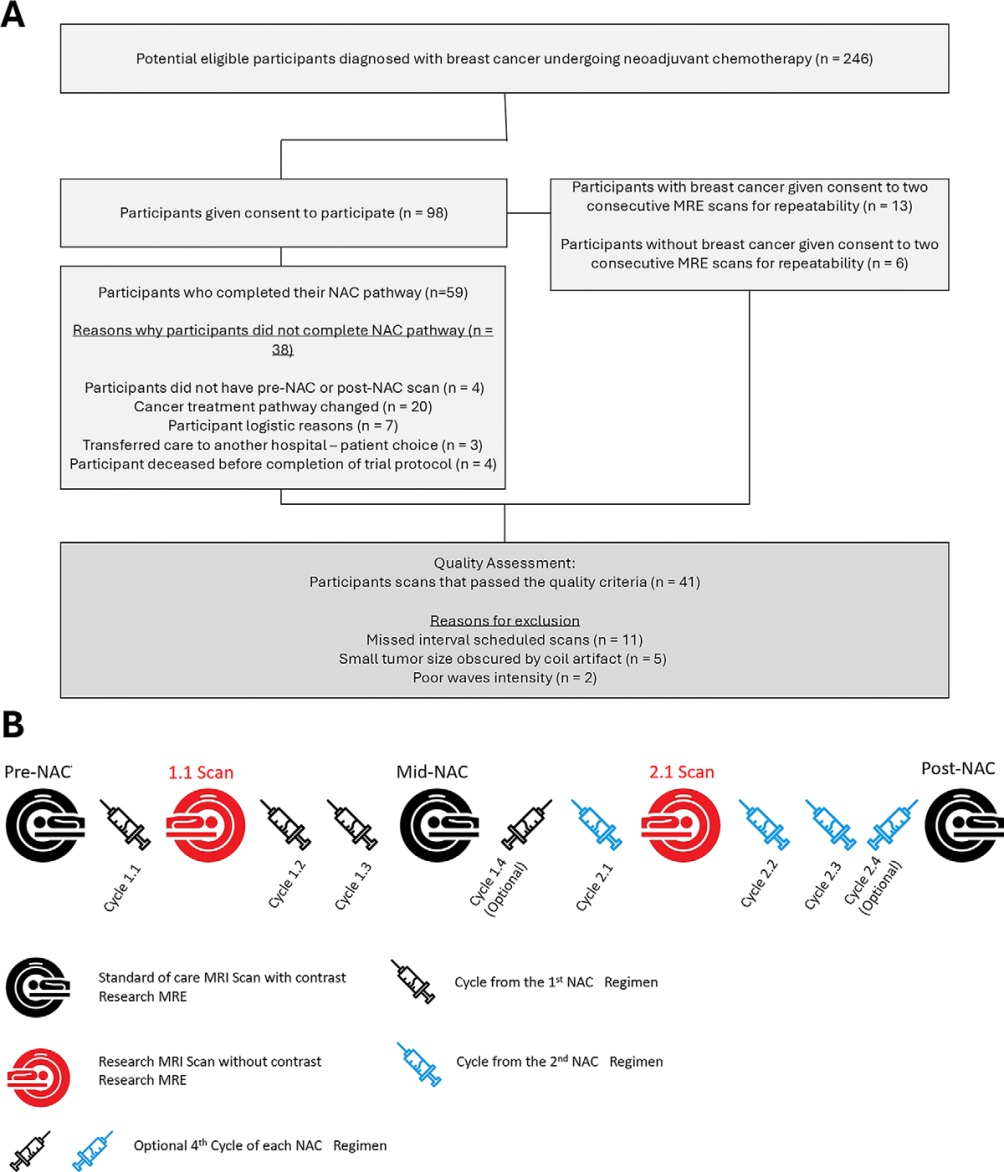
Flowchart and study pathway. **(A)** Flowchart of participant inclusion. Ultimately, datasets from 41 participants who fulfilled all necessary criteria (ie, all scans performed throughout neoadjuvant chemotherapy [NAC], all scans with sufficient quality, and post-NAC histopathologic analyses available) were included in this analysis. **(B)** Participants received three or four cycles of the first regimen and three or four cycles of the second regimen prior to post-NAC surgical intervention. In total, five MRI and MR elastography (MRE) sessions were interlaced with the NAC regimen.

This single-arm phase II prospective study (conducted between September 2020 and August 2023) was approved by an independent review board (reference nos. 16/LO/1303, NCT03238144), and all participants provided written informed consent. Inclusion criteria were female participants aged 18 years or older with invasive breast cancer who were scheduled to undergo NAC and able to provide written informed consent. Exclusion criteria were prior ipsilateral breast cancer, inability to provide written informed consent, and contraindications for MRI. Participant demographics, tumor characteristics, treatment regimens, and radiologic and pathologic responses were recorded. A flowchart of patient inclusion is shown in Figure 1A. All participants underwent surgery after NAC. The breast specimens were assessed for response to NAC using the MD Anderson RCB score (4). The RCB score was categorized as RCB-0 (pCR, RCB = 0), RCB-I (0.5 < RCB ≤ 1.36), RCB-II (1.36 < RCB ≤ 3.28), and RCB-III (RCB > 3.28). This classification allowed for categorizing patients into those who achieved a pCR or partial response (RCB-I, -II, or -III).

MRI plus MRE scans were performed before treatment (pre-NAC), halfway through treatment (mid-NAC), and at the end of treatment (post-NAC) (Fig 1B). Two additional MRE-only scans were performed after the first cycle of the first NAC regimen (postcycle 1.1) and after the second NAC regimen (postcycle 2.1). With the current study aims (ie, gauging pathologic response and identifying potentially early resistance or response), this article focuses on the following three time points: pre-NAC, postcycle 1.1, and post-NAC. In all, 41 participants underwent all five scans, including three clinical DCE MRI acquisitions.

### MRE Hardware and Sequencing

**Figure 2:**
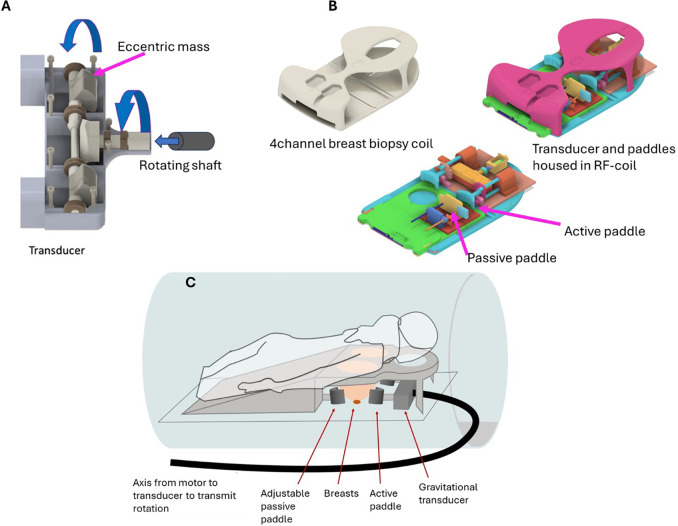
Gravitational transducer (GT) setup for the breast MRI coil. **(A)** The GT-based MR elastography breast setup consists of two eccentric masses that rotate around an axis that is oriented right-left. The flexible shaft that transmits the rotations from the motor to the transducer arrives from the head direction and connects to the transducer via a bayonet connection. **(B)** The entire GT setup is incorporated into the four-channel biopsy coil from Siemens. It hosts the transducer as well as active and passive paddles. The passive paddles are movable in the feet-head direction to ensure that the breasts have proper mechanical contact with the active paddles, which are fixed to the GT and only vibrate and cannot be moved. **(C)** Sketch of the entire setup showing the patient, the paddles, the GT, and the rotating flexible shaft entering from the head side. RF = radiofrequency.

Scans were performed using a MAGNETOM Aera 1.5-T MRI system (Siemens Healthineers). Standard MRI protocols were acquired using T1-weighted, T2-weighted, diffusion-weighted, and DCE sequences. To enable MRE, the gravitational transducer (26) was integrated into the Siemens four-channel breast biopsy radiofrequency coil (Fig 2). Participants were positioned prone and head first with the breasts placed in the designated openings of the radiofrequency coil. The gravitational transducer had two rotating eccentric masses generating longitudinal vibrations in feet-head direction (Fig 2A). Mechanical vibrations were transmitted in the breasts through active paddles. Passive paddles, adjustable in the feet-head direction, were used to ensure good mechanical contact between the breasts and active paddles (Fig 2B). The gravitational transducer was connected to the driving motor unit via a 9m flexible rotating axis (Fig 2C). MRE data were acquired using a gradient-echo sequence (eXpresso) (27) at 36 Hz with fractional motion-encoding gradients at 20 mT/m. Imaging parameters were as follows: 16 sections; 3-mm isotropic voxel size; 128 × 128 acquisition matrix; and an in-plane generalized autocalibrating partial parallel acquisition acceleration factor of 2, resulting in a field of view of 384 × 384 × 48 mm^3^; echo time of 9.2 msec; repetition time of 222 msec (16 × 13.9 msec); and a flip angle of 25°. The field of view was centered on the tumor core, which was identified using anatomic scans. MRE acquisitions took approximately 7 minutes, with total acquisition times of 25 minutes for clinical scans and 16 minutes for research scans only.

### MRE Postprocessing

MRE data were processed according to Sinkus et al (28). In short, applying the curl operator removes contributions from compressional waves from the total wave field. Spatial derivatives necessary to solve the wave equation were calculated in Fourier space to improve quality and robustness. An 11th-order Blackman-Harris filter was applied to suppress noise. Before Fourier transformation, the wave field was smoothed with a Gaussian filter (3 × 3 × 3 pixels stealth, 0.75 pixels sigma). The final three complex-valued equations (Helmholtz-type) are solved for the wave vector 
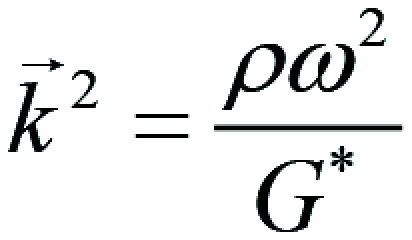
 through a *χ*^2^ minimization approach, with *ρ* the density, *ω* the circular frequency, and G* = G′+ iG″ the complex-valued shear modulus with *G′* elasticity and *G″* viscosity (in kilopascals). From G* the phase angle 
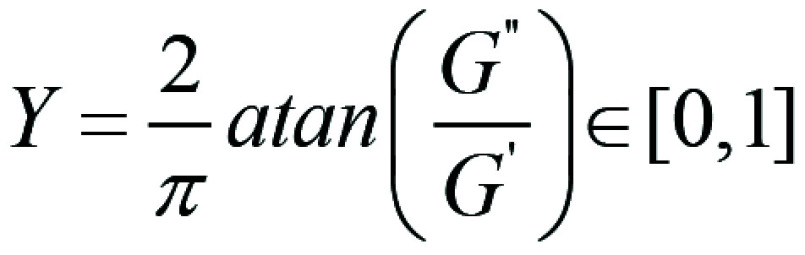
 is derived.

### MRE Biologic Markers and Statistical Analysis

Regions of interest (ROIs) were drawn by a consultant radiologist (A.M.) with more than 15 years of experience with breast MRI. Guided by standard diagnostic scans, ROIs were placed accordingly on the MRE magnitude images without knowledge of the underlying maps depicting biomechanics. ROIs were drawn over the tumor area for the pre-NAC and postcycle 1.1 MRE, excluding the region of the localization marker clip (see Fig S1). Because after NAC there is frequently no discernible tumor visible anymore (only two participants had discernible tumors), the initial tumor footprint ROI from before NAC was used as a landmark for ROI placement. A more detailed description is provided in Appendix S1.

Biologic markers were quantified within the corresponding ROIs via either the mean value of the distribution in case of tumor elasticity or the mean peak value of a Gaussian or Landau fit for the phase angle (depending on the lowest χ^2^ of the fit, correspondingly). In addition to absolute values, ratios of biologic markers were investigated to more easily identify trends among participants and study their temporal evolution throughout therapy. Specifically, we investigated the tumor stiffness ratio (TSR) 
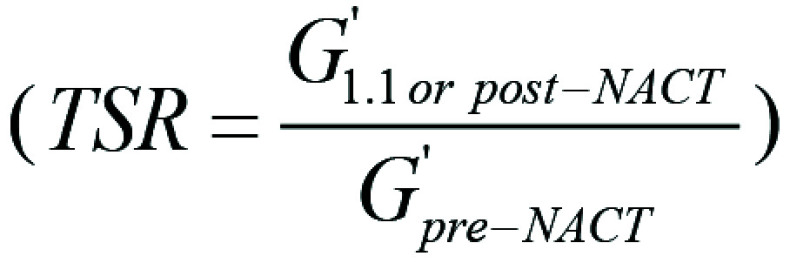
 and phase angle ratio (PAR) 
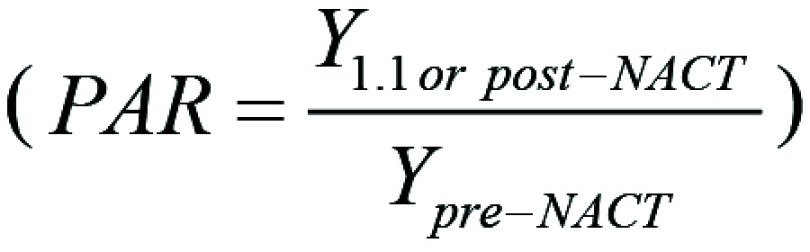
 with their corresponding normalization relative to before NAC.

The TSR is defined as the ratio of elasticity measured after NAC (post-NAC) to the elasticity measured before the treatment (pre-NAC). A TSR greater than 1 indicates a decrease in elasticity over time and that the tissue is less elastic at post-NAC compared with at pre-NAC. Conversely, a TSR less than 1 suggests an increase in elasticity before NAC compared with after NAC. If the elasticity remains unchanged, the TSR will equal 1. The expected range is between 0.1 and 2, where in the most extreme cases the elasticity has a significant drop or a doubling of the initial elasticity observed prior to NAC.

The PAR is defined as the ratio of the phase angle at postcycle 1.1 to the phase angle measured at pre-NAC. A PAR greater than 1 indicates a decrease in phase angle at 1.1 compared with before NAC. A PAR smaller than 1 shows a phase angle increase at 1.1 compared with before NAC. For a PAR of 1, the phase angle remains unchanged. Similarly to the TSR, the more extreme cases could decrease or elevate the ratio to 0.1 or 2, respectively.

Data quality criteria were local shear wave amplitude (Atot ≥ 95 μm), nonlinearity (<35%, which indicates how much the phase signal intensity deviates from a perfect sinusoidal), and the wave signal-to-noise ratio (>3) expressed by the ratio of the magnitude of the wave’s rotation over its divergence. Additionally, scans were rejected if the number of exploitable voxels within an ROI dropped below 120 pixels whereby rendering the corresponding biologic markers not statistically reliable (Fig S2). Any tumor region that did not meet these criteria on average was excluded from the analysis.

Independent samples *t* tests or Wilcoxon signed rank tests were used to compare complete and partial responders, depending on normality (Shapiro-Wilk test). Repeatability was assessed using Bland-Altman analysis, intraclass correlation coefficients, and the repeatability coefficient (the full protocol is in Appendix S1). The predictive ability of MRE-derived parameters were assessed using the receiver operating characteristic curve for DCE MRI, MRE, and a combined approach of DCE MRI and MRE. The receiver operating characteristic analysis resulted in area under the curve, specificity, and sensitivity. In the combined approach a cutoff value was used which was based on the repeatability coefficient. Significance was set at a *P* < .05. All statistical analyses were conducted using SPSS (version 29.0.2.0 [20]; IBM SPSS statistics).

## Results

### Participant Characteristics

All MRE scans were assessed for quality, as previously described. Participants who did not undergo the complete imaging protocol or failed to meet all criteria were excluded from the analysis. Ultimately, we identified 41 participants who met all criteria for inclusion into the study (Fig 1A) (mean age, 47 years ± 12.5 [SD]; all participants were female). Overall cohort demographics and details regarding tumor types and corresponding receptor status for each included participant are shown in Tables 1, 2, and S1. Histopathologic analysis was used to classify each participant into partial responders or complete responders.

**Table 1:**
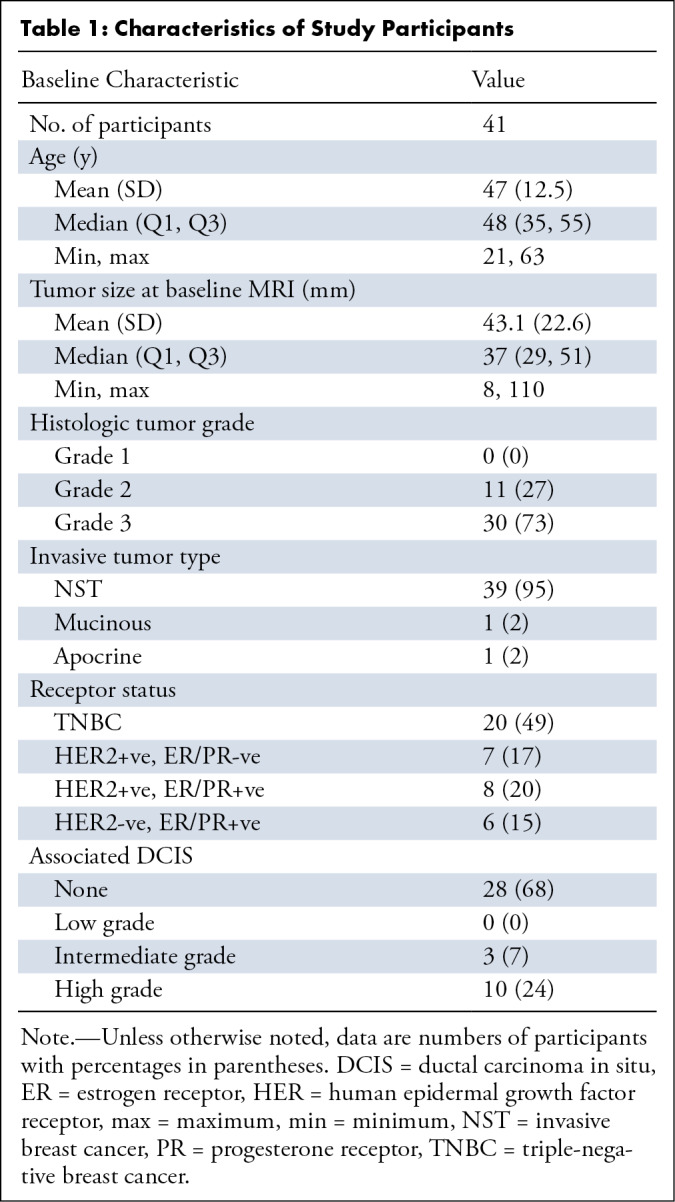
Characteristics of Study Participants

**Table 2:**
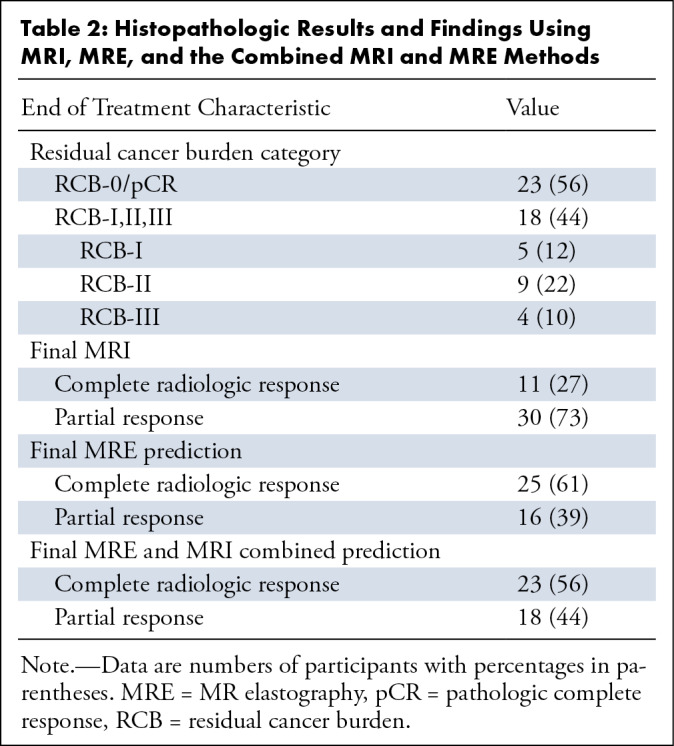
Histopathologic Results and Findings Using MRI, MRE, and the Combined MRI and MRE Methods

Twenty-three of the 41 participants (56%) achieved pCR. The pathologic response depended on tumor type (*X*^2^ test; *P* = .004), with pCR more likely in triple-negative breast cancer and human epidermal growth factor receptor 2–positive and hormone-negative breast cancers. The pathologic response was also dependent on tumor grade (*X*^2^ test; *P* = .02), with grade 3 tumors more likely to have pCR than grade 2 tumors (Table S2).

### Biomechanics and Association with Response or Resistance

**Figure 3:**
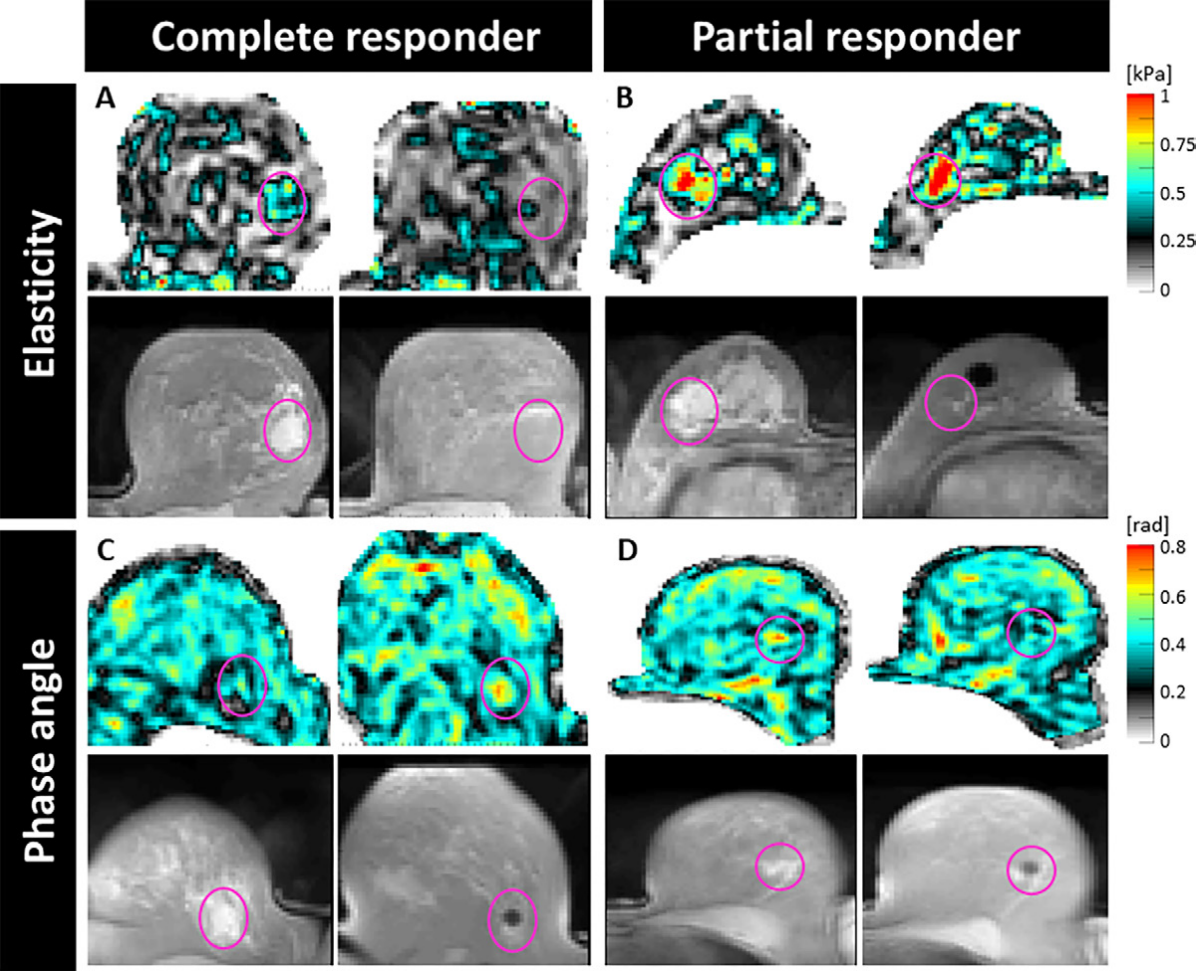
Axial elastograms with corresponding T2-weighted images as examples for biomechanical changes in the tumor region. **(A, B)** Stiffness evolution for a responder (aged 72 years) and partial responder (aged 28 years) from before NAC (left) to after NAC (right), respectively. Response appears to lead to a relative drop in tumor bed stiffness, while resistance leads to a corresponding increase. **(C, D)** Phase angle evolution for a responder (aged 47 years) and partial responder (aged 32 years) from before NAC (left) to time point 1.1 (right), respectively. Response appears to lead to a relative increase in phase angle within the tumor bed, while resistance leads to a corresponding drop.

**Figure 4:**
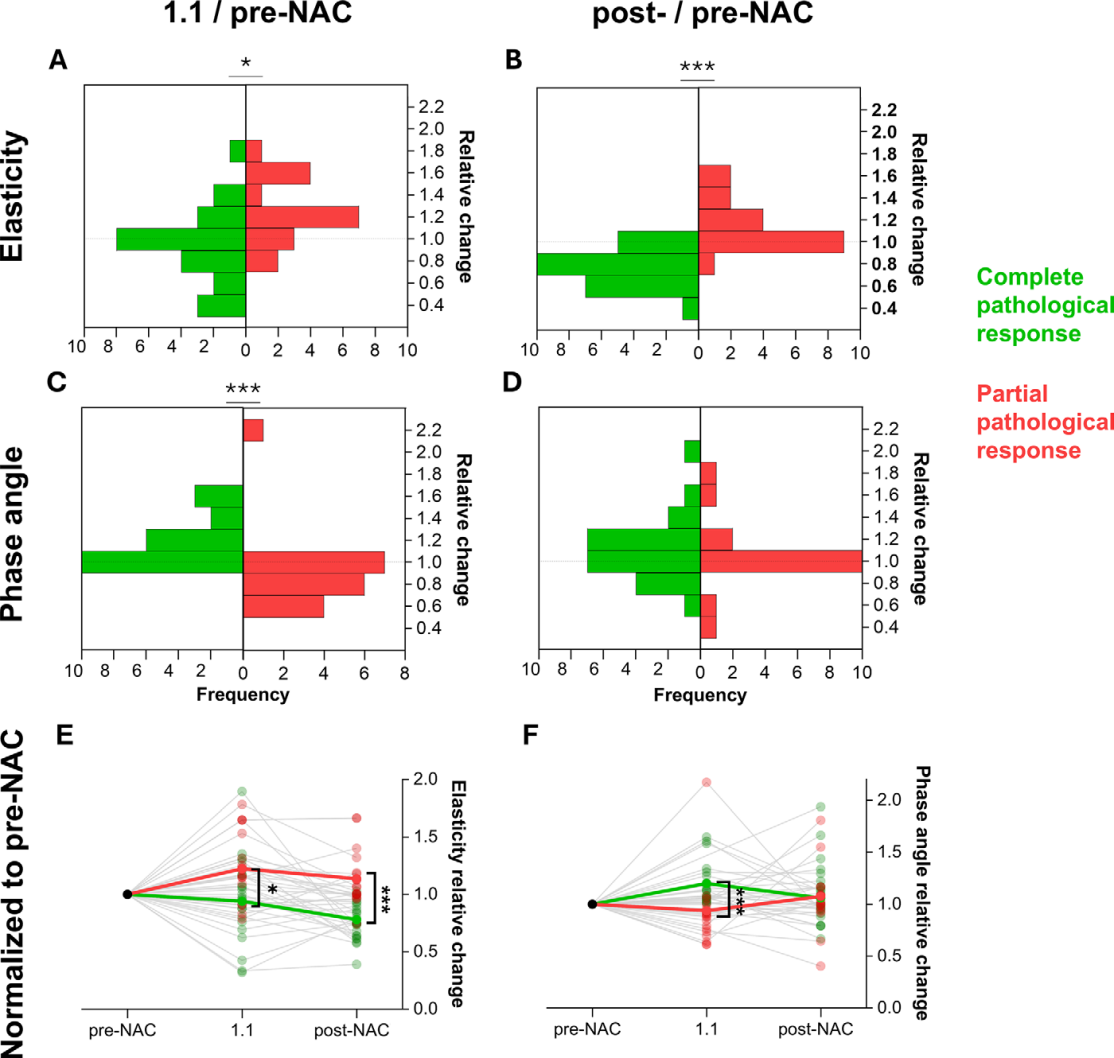
Changes in biomechanics at different time points. **(A–D)** Histograms of the relative change for elasticity for cycle 1.1 and following neoadjuvant chemotherapy (NAC) normalized to before NAC **(A, B)** and phase angle for cycle 1.1 and following NAC normalized to before NAC **(C, D)**, respectively. **(E, F)** Stem plots are shown for the elasticity **(E)** and phase angle **(F)** normalized to before NAC for postcycle 1.1 and following NAC. Note that these measures originate before NAC and after NAC from the tumor footprint while at 1.1 from the tumor region of interest. Green indicates all participants with complete pathologic response and red indicates all participants with partial pathologic response. * *P* < .05, *** *P* < .001.

The absolute values of elasticity and phase angle for all participants as a function of the NAC regimen are consolidated within Table 3 and Figure S3, with the respective results of the post hoc analysis shown in Table S3. Figure 3 shows maps of elasticity and phase angle at the pre-NAC, post-NAC, and postcycle-1.1 acquisitions for four selected participants who demonstrated response or resistance. These results are quantified in Figure 4 for the entire cohort, which presents TSR and PAR ratios for the relevant time points.

**Table 3:**
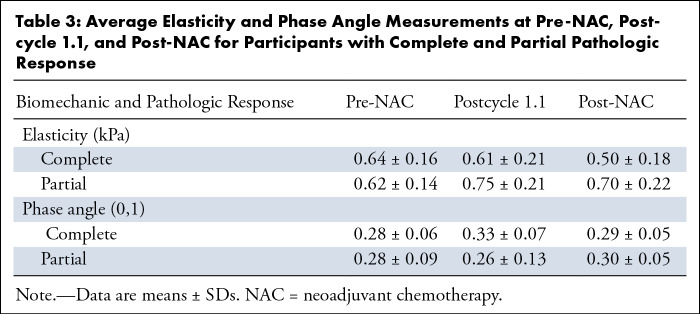
Average Elasticity and Phase Angle Measurements at Pre-NAC, Postcycle 1.1, and Post-NAC for Participants with Complete and Partial Pathologic Response

**Figure 5:**
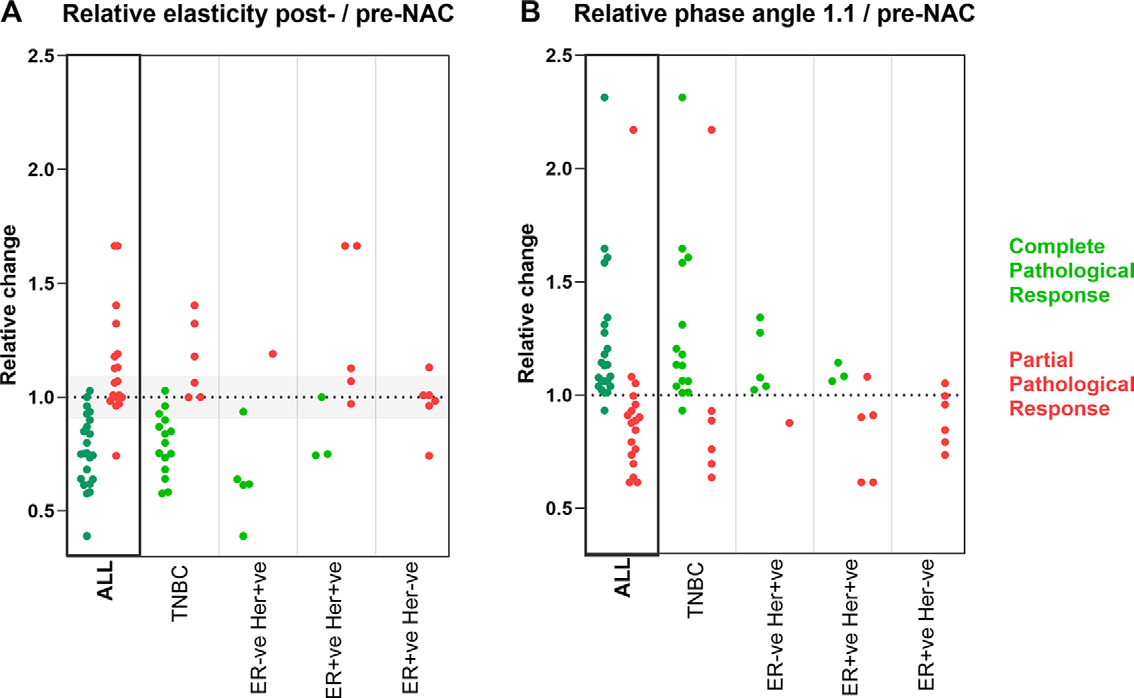
Tumor stiffness and phase angle gauge pathologic response at end–neoadjuvant chemotherapy (NAC) and early at 1.1, respectively. **(A)** The change in tumor bed stiffness at post-NAC relative to pre-NAC is shown for all participants (left) and consecutively as a function of the individual receptor status of the patient. Green circles indicate participants with pathologic complete response, while red circles indicate participants with partial pathologic response (as established from histopathologic analysis after surgery). A stable or rising ratio is indicative of resistance, while a drop in this ratio is indicative of response. As expected, most responders belong to the triple-negative breast cancer (TNBC) receptor status group. The horizontal gray region indicates the repeatability coefficient. **(B)** The change in phase angle within the tumor region after the first cycle (1.1) relative to pre-NAC is shown for all participants (left) and consecutively as a function of the individual receptor status of the patient. Here, a drop in phase angle is indicative of resistance while a rise is indicative of response.

Regarding TSR, response was significantly associated with a drop in relative tumor stiffness at time point 1.1 (Fig 4A) and post-NAC (Fig 4B) acquisitions. Quantitatively, the ratio of elasticity between pre- and post-NAC acquisitions showed a prominent decrease for complete responders (TSR, 0.76 ± 0.16) and an increase for partial responders (TSR, 1.14 ± 0.24). A comparison of both ratios showed a significant difference (*P* < .001) (Fig 5A). As mentioned above, the TSR at time point 1.1 also showed a significant difference between complete and partial responders (complete: TSR, 0.95 ± 0.35 vs partial: TSR, 1.25 ± 0.30; *P* = .007). Figure 5 additionally shows the results as a function of receptor status with, as expected, most pCR belonging to the triple-negative breast cancer and human epidermal growth factor receptor 2–positive and hormone-negative breast cancer groups.

A clear drop in relative phase angle within the tumor region was observed for partial responders compared with complete responders at time point 1.1 (1.23 ± 0.31 vs 0.91 ± 0.34; *P* < .001), with no evidence of a difference between groups following NAC (1.10 ± 0.29 vs 1.27 ± 0.70; *P* = .47) (Figs 4, 5).

Figure S3 shows the elasticity and phase angle throughout NAC. Figure S4 shows the elasticity and phase angle prior to NAC as a function of Breast Imaging Reporting and Data System density scores. Furthermore, Figure S5 shows the correlation between PAR and TSR for complete pathologic responders and partial responders.

### Repeatability

One participant’s repeatability data had to be excluded due to a mechanical failure of the MRE system, leaving 12 participants for the repeatability analysis. The stiffness and phase angle measurements of these participants showed excellent repeatability limits of agreement for elasticity (limits of agreement: -14%, 12%) and for the phase angle (limits of agreement: -12%, 19%) in the tumor. A graphical representation can be found in Figure S6 for the tumorous tissue and apparent healthy tissue taken from the contralateral side and healthy volunteers who underwent a repeatability protocol with repositioning. Repeatability analysis showed an intraclass correlation coefficient of 0.99 (95% CI: 0.95, 0.99) and a repeatability coefficient of 8.3% for elasticity and an intraclass correlation coefficient of 0.88 (95% CI: 0.59, 0.96) and repeatability coefficient of 5% for the phase angle.

### Combined Approach

**Figure 6:**
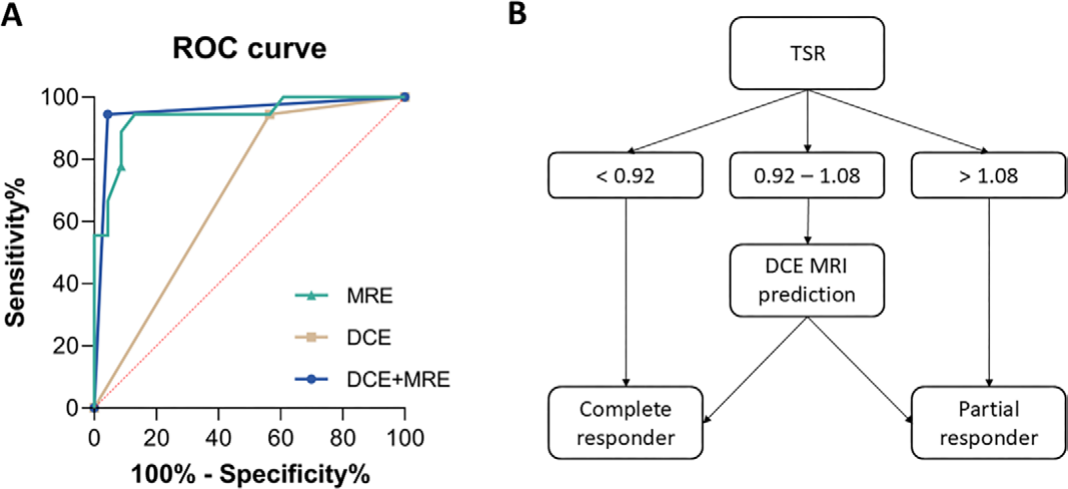
Receiver operating characteristic (ROC) analysis for different predictors of pathologic response. **(A)** ROC curve for different approaches to predict complete pathologic response: tumor stiffness ratio (TSR; green), classic MRI using dynamic contrast-enhanced (DCE) MRI (beige), and dual-approach DCE MRI and MR elastography (MRE; blue). **(B)** Proposed decision pathway to combine DCE MRI at post-NAC and TSR for predicting pathologic response.

We compared the current state-of-the-art approach using DCE MRI following NAC for predicting pCR with TSR when using a cutoff value of 1. The corresponding receiver operating characteristic curves are presented in Figure 6A. TSR outperformed the DCE MRI–based approach in terms of area but is less sensitive. DCE MRI had a sensitivity of 94% (95% CI: 74, 100) and a specificity of 44% (95% CI: 26, 63) alone, while TSR yielded a sensitivity of 72% (95% CI: 49, 88) and a specificity of 91% (95% CI: 73, 98). To foster the complementary information provided by both methods, a combined approach was investigated. This relies on determining whether the TSR value surpasses the repeatability coefficient threshold (ie, 8.3%), indicating discernible variances (Fig 6B). In cases where TSR was beyond the interval excluded by the repeatability coefficient (ie, TSR < 0.92 or TSR > 1.08), it was used to predict pCR. Otherwise, when TSR fell into the interval of statistical insignificance (TSR∈ [0.91,1.09]), the classic DCE MRI–based prediction for pCR was used. This dual-biomarker approach provided an elevated specificity of 96% (95% CI: 79, 100) while maintaining a high sensitivity of 94% (95% CI: 74, 100) (Fig 6A). The respective area under the receiver operating characteristic curve values were 0.69 (0.53–0.85) (DCE MRI), 0.85 (0.86–1.0) (TSR), and 0.95 (0.87–1.0) (DCE MRI plus MRE). Results are compiled in Table 4.

**Table 4:**
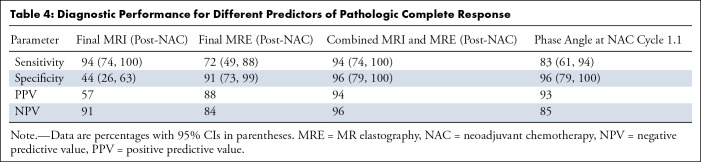
Diagnostic Performance for Different Predictors of Pathologic Complete Response

## Discussion

This prospective study evaluated the use of biomechanics (elasticity and phase angle), quantified noninvasively via MRE, to assess response in individuals with breast cancer undergoing NAC. Elasticity alongside standard clinical DCE MRI scans resulted in an increased specificity (44% to 96%) for the prediction of pCR, as assessed by histopathologic analysis, which could potentially be of importance for the de-escalation of surgical intervention. Furthermore, the phase angle showed the possibility of early prediction of pCR (1.23 ± 0.31 vs 0.91 ± 0.34; *P* < .001). Analysis of these two parameters throughout the time course of NAC provided several insights into therapy-induced changes in biomechanics within the tumor and tumor microenvironment.

First, we found that the replacement of tumorous tissue within the initial tumor footprint by healthy tissue led to drastic changes in mean stiffness upon completion of NAC. Analysis showed that participants with pCR were more likely to exhibit a decrease in stiffness within the tumor footprint following NAC compared with those with partial response. This finding suggests that normal healthy tissue regenerated within the area of the tumor footprint tends to be softer than the initial cancerous tissue. Notably, use of TSR demonstrated a significant association with pCR, and thus, appears to be strongly indicative for pCR.

Second, the relative change in phase angle after the first cycle seems to be highly predictive for pCR, with a drop in relative phase angle indicative of resistance. It is widely noted that resistance leads to fibroblast-induced collagen deposition (29). This theoretically should lead to a drop in phase angle due to the addition of mainly purely elastic composites, which is in accordance with our observation. Fibroblast-induced collagen deposition should concomitantly lead to an increase in stiffness, which is equally observed in our data. Shortly after the first cycle of NAC, there is a disruption of the tumor and its microenvironment resulting in reduction in tumor cell proliferation, increased vascular permeability, edema, tumor necrosis, and inflammation. This results in an increase in osmotic pressure leading to an increase in interstitial fluid pressure, thereby increasing viscosity. This would result in a change in the phase angle at time point 1.1, predicting for complete or partial responders following NAC. This early change is also observed in measurement of elasticity (tumor stiffness) at 1.1 relative to before NAC. While this difference is less prominent than at the end of NAC, it does indicate early tissue alterations regarding biomechanics that correlate to response or resistance, and the rise in elasticity seen for partial responders matches the observed clear drop in phase angle. In addition, recent evidence (30) using transcriptome profiling coupled with histopathologic analyses showed that the first cycle of NAC induced an immune stimulatory response in the microenvironment with upregulation of inflammatory signatures in tumors that was independently associated with a pCR. Furthermore, the first cycle of NAC induced downregulation of cell-cycle genes exemplified by pathways related to cell growth and proliferation.

Third, biomechanics showed higher specificity over the current reference standard clinical MRI using DCE MRI for gauging pCR, with a slightly lower sensitivity. However, a combined biomarker approach of MRE and DCE MRI improved specificity, increasing from 43.5% (DCE MRI alone) to 95.7% (MRE and DCE MRI) while maintaining the high sensitivity of DCE. With the intensively discussed topic of de-escalation of surgery in trials, such as in the phase II trial conducted by Kuerer et al (12), MRE may have an additional role alongside MRI as a noninvasive method to accurately identify patients who have complete clinical response.

There are limitations to our study that should be considered. While the ability to use phase angle as a potential predictor for response or resistance after one cycle of chemotherapy is intriguing, the precise biophysical and biochemical mechanisms underlying the disparity in PAR between complete and partial response remain currently conjectural. Furthermore, response and resistance are gauged via histopathologic analysis after NAC, and this information may not necessarily be representative of changes in biomechanics occurring after one cycle of therapy. Currently, the clinical protocol does not include DCE MRI or additional core biopsies for the quantifications performed at time point 1.1. While findings appear promising, this early patient cohort precludes definitive conclusions. Last, exploring MRE within clinical routine necessitates the incorporation of a mechanical transducer into the radiofrequency coil. Our gravitational transducer setup was well tolerated by all participants with no adverse effects. Additionally, the presence of the transducer paddles fixating the breasts led to reduced motion artifacts than in standard clinical routine data.

To summarize, we have integrated gravitational transducer–based MRE into the clinical workflow of NAC follow-up for individuals with breast cancer and presented three main results: *(a)* a drop in relative tumor bed stiffness observed following NAC was indicative of pCR, *(b)* the combination of this biomechanics-based imaging biomarker with established DCE significantly increased specificity while maintaining the high sensitivity of DCE MRI alone for the identification of complete responders following NAC, and *(c)* a relative change in biomechanical phase angle as early as after the first cycle of NAC was significantly associated with pCR. All three findings warrant further in-depth investigation, including larger (multicenter) cohorts in conjunction with reference standard data through sequential tumor biopsies during NAC. Overall, this study demonstrates the pertinence of biomechanics quantified through noninvasive MRE to provide additional complementary information to clinical decision-making in the context of NAC.
